# Analysis and prediction of condylar resorption following orthognathic surgery

**DOI:** 10.1038/s41598-024-81148-w

**Published:** 2025-01-03

**Authors:** Pieter-Jan Verhelst, Sigrid Janssens, Harold Matthews, Giacomo Begnoni, Peter Claes, Eman Shaheen, Hilde Peeters, Constantinus Politis, Reinhilde Jacobs

**Affiliations:** 1 OMFS IMPATH Research Group, Department of Imaging and Pathology, Faculty of Medicine, KU Leuven, Leuven, Belgium; 2 Department of Oral and Maxillofacial Surgery, University Hospitals Leuven, Leuven, Belgium; 3 Department of Public Health and Primary Care, Gerontology and Geriatrics, KU Leuven, Leuven, Belgium; 4 Department of Human Genetics, KU Leuven, Leuven, Belgium; 5 Medical Imaging Research Center, University Hospitals Leuven, Leuven, Belgium; 6 Facial Sciences Research Group, Murdoch Children’s Research Institute, Parkville, Australia; 7 Orthodontics Research Unit, Department of Oral Health Sciences, Faculty of Medicine, KU Leuven, Leuven, Belgium; 8 Department of Orthodontics, University Hospitals Leuven, Leuven, Belgium; 9 Department of Electrical Engineering, ESAT/PSI, KU Leuven, Leuven, Belgium; 10 Department of Human Genetics, University Hospitals Leuven, Leuven, Belgium; 11 Department of Dental Medicine, Karolinska Institutet, Stockholm, Sweden

**Keywords:** Orthognathic surgery, Condylar resorption, Condylar remodeling, Bilateral sagittal split osteotomy, Temporomandibular joint, Diseases, Medical research, Risk factors, Signs and symptoms

## Abstract

Condylar resorption is a feared complication of orthognathic surgery. This study investigated condylar resorption in a cohort of 200 patients This allowed for a powerful update on incidence and risk factors. 9.5% of patients developed resorption. These patients had on average, 17% volume loss with 3.9 mm ramal height loss and 3.1 mm posterior mandibular displacement. 2% of patients had bilateral resorption. Univariable analysis identified a younger age, a bimaxillary + genioplasty procedure, larger mandibular advancements, upward movements of the distal segment, a higher counterclockwise pitch of the distal segment, smaller preoperative condylar volumes and a higher anterior/posterior lower facial height ratio as risk factors on a patient level. Univariable analysis on a condylar level also identified compressive movements of the ramus and a higher mandibular plane angle as risk factors. Using machine learning for the multivariable analysis, the amount of mandibular advancement was the most important predictor for condylar resorption. There were no differences in preoperative mandibular, ramal or condylar shape between patients with or without resorption. These findings suggest condylar resorption may be more common than thought. Identifying risk factors allows surgical plans to be adjusted to reduce the likelihood of resorption, and patients can be more selectively screened postoperatively.

## Introduction

**Fig. 1:**
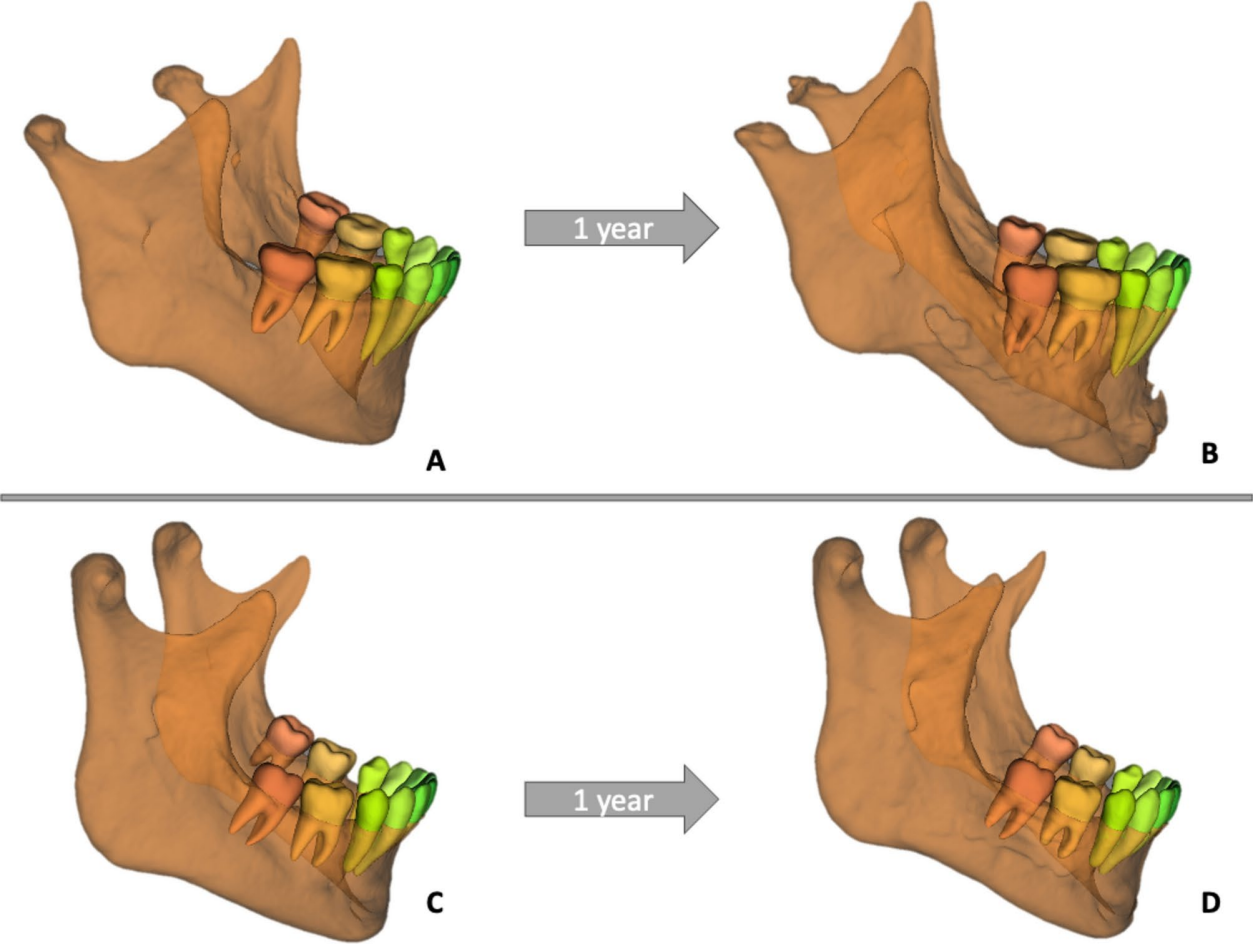
Examples of condylar resorption and condylar remodeling 1 year after BSSO. **A** shows a preoperative mandible of a patient that will develop resorption. **B** shows the mandible 1 year after the BSSO with bilateral condylar resorption. **C** shows a preoperative mandible of a patient that will have normal condylar remodeling. **D** shows the mandible 1 year after the BSSO with no major changes in condylar form.

Skeletal dysgnathias and their resulting malocclusions are often characterized by functional problems that significantly impact patients’ quality of life, affecting their ability to eat, speak, or breathe^1,2^. In addition, these patients may have aesthetic concerns due to facial asymmetry or disproportional jaw sizes^3^. Orthognathic surgery aims to correct these problems by normalizing the position and alignment of the jaws, improving the bite and restoring proper function and facial balance. The most performed orthognathic surgery procedure is the bilateral sagittal split osteotomy (BSSO) introduced by Obwegeser in 1953^4,5^. The technique was modified by Epker, Hunsuck, Dal-Pont and Wolford to be the workhorse of current-day orthognathic surgery^6^. It is a safe and reliable procedure in which the proximal condylar-bearing segments are seated in the fossa, and the distal teeth-bearing segment is repositioned and fixed into a new occlusion-guided position, either on its own or in combination with the maxilla through a Le Fort osteotomy. The BSSO causes biomechanical stress in the temporomandibular joint (TMJ) due to intra-operative and postoperative factors^7^. During surgery, periosteal stripping, performing the split, mobilizing the condyle in the fossa and fixing the new position of distal segment using osteosynthesis material are stressors for the joint as they cause some degree of biological trauma to the condyle^7^. In the postoperative phase such factors are prolonged condylar compression in the fossa, muscular traction on the proximal segment and the use of postoperative elastics^7^. These are factors that are inherently part of the BSSO and can vary in extent. These factors lead to a degree of biomechanical stress in the joint which induces condylar remodeling, a physiological response expected in each patient that has surgery. However, in some orthognathic surgery patients, this process surpasses the physiological adaptive remodeling capacity^8^. In these cases, condylar resorption occurs (Fig. 1)^9–11^. This complication in orthognathic surgery is characterized by a sudden volume loss of the condyle, resulting in ramal height loss and posterior displacement of the mandible^12,13^. Postoperative condylar resorption is clinically characterized by the reoccurrence of a malocclusion (class 2 malocclusion and anterior open bite) and facial changes (retrognathia of the mandible and increased anterior lower facial height)^14,15^. In the acute resorption phase, joint function can be limited and characterized by pain and a limited range of motion^16^.

This makes condylar resorption a feared complication as it destroys the result of a long treatment of combined orthodontics and orthognathic surgery. Incidence numbers are heterogeneous, ranging from 1 to 31% of orthognathic surgery patients^15^. Most of these numbers are based on studies using 2D assessment of the condyle and skeletal relapse. Over the past years, results on the 3D assessment of condylar remodeling and resorption have emerged^17,18^. Although these findings were necessary to understand condylar resorption better, they had some significant limitations, such as partial use of the 3D information from cone-beam computed tomography (CBCT) follow-up imaging and limited patient numbers. The establishment and incorporation of virtual surgical planning and 3D follow-up, combined with new techniques such as automated segmentation and statistical shape analysis, allow larger study groups, and leverage the full potential of 3D information in our CBCT scans. Also, machine learning allows for more powerful analysis methods in prediction modeling and identifying risk factors in datasets with low observations of pathological cases and a high number of potential predictors. By combining these new methods, the limitations of previous studies can now be addressed.

This study aims to investigate the occurrence of condylar resorption in a large cohort of orthognathic surgery patients receiving either only a BSSO or a BSSO combined with a Le Fort I osteotomy. As a secondary objective, potential risk factors contributing to condylar resorption development are investigated, and a prediction model will be built. Finally, the preoperative mandibles of patients with and without resorption will be evaluated for differences in shape.

## Materials and methods

### Data acquisition and image-preprocessing

This is a prospective observational study using the LORTHOG registry (prospectively collected data on follow-up of orthognathic surgery patients from the Department of Oral & Maxillofacial Surgery, University Hospital Leuven, Belgium) approved by the local ethical committee (B322201526790) ensuring that all experiments were performed in accordance with relevant guidelines and regulations. Informed consent was obtained from all subjects and/or their legal guardian(s). Patients who had surgery from May 2017 to December 2019 and were included in the database were screened for inclusion criteria: (1) having a BSSO whether in combination with genioplasty or Le Fort surgery and (2) having at least one year of follow-up with CBCT-imaging. The exclusion criteria were (1) patients with craniofacial syndromes or cleft lip and/or palate and (2) patients needing revision orthognathic surgery. No exclusion based on angle class was made, so both class two as class three patients were included. This study included two hundred consecutive patients fulfilling these criteria in the set time frame. All patients were operated by the same surgical team, using an Hunsuck split and rigid fixation with two mini-osteosynthesis plates with monocortical screws on each side.

The following variables were extracted from the registry: age, angle class of malocclusion, type of surgery, sex, body mass index (BMI), maximal interincisal mouth opening preoperatively, preoperative TMJ sounds and pain. Sounds and pain were recorded as a yes/no variable during the preoperative work-up. The 6-week preoperative (T0), 1-week postoperative (T1) and 1-year postoperative (T2) CBCT scans were used for the 3D analysis. Each scan was taken with a Newtom VGi evo device (Cefla, Imola, Italy) with the scanning parameters of FOV 24 × 19 cm, voxel size 0.3 mm3, 110 kV and 4.3 mA. Patients were scanned at all time points with a thin wax bite positioning the mandible in centric relation. The digital imaging and communication in medicine (DICOM) files of each scan were exported and loaded into an artificial intelligence (AI) driven segmentation platform (Virtual Patient Creator, Relu Bv, Leuven, Belgium) allowing precise segmentation of the maxillofacial skeleton. The use of the Virtual Patient Creator has been validated with excellent inter- and intra-operator reliability^19^. This resulted in stereolithography (STL) models of the preoperative maxilla and mandible and the postoperative mandible that were available for the 3D analysis.

### 3D analysis

Surgical movements of the proximal and distal mandibular segments (T0 vs T1) and the relapse of the distal mandibular segment (T1 vs T2) were calculated by means of a step-wise voxel-based registration tool in Amira software (v6.7, Thermo Fisher Scientific, Merignac, France) that has been previously validated by Shujaat et al.^20,21^. The surgical movements of all segments were determined by importing the DICOM data of T0 and T1 time points and first registering them on the cranial base. Next, the T1 data was transformed into the T0 position using voxel-based registration by delineating a volume of interest in both T0 and T1. For the distal segment, this was the mandibular body unaffected by any osteotomy, such as a genioplasty. For the proximal region, a modified ramus, as described by Verhelst et al.^22^ was used. In a next step, the mandibular distal and left and right proximal segments were imported as STL files. A singular value decomposition algorithm was applied to the transformation matrices from the voxel-based registration of all three segments to calculate the difference in the six degrees of freedom: three translational (anteroposterior, superoinferior and mediolateral) and three rotational (pitch, roll and yaw) movements^23^. The resulting numbers are the surgical movements of the segments between T0 and T1. The same procedure with the same volume of interest was performed for the distal segment between T2 and T1 to assess the skeletal relapse of the mandibular body.

Linear and angular measurements were made on the preoperative 3D models using 3-Matic (Materialise, Leuven, Belgium): the anterior lower facial, ramal height, and mandibular plane angle. The ramal height was also measured on the T2 model. The C-plane was constructed on the T0 mandible on each side separately to delineate the caudal border of the condyle in order to calculate condylar volumes^11^. To achieve the same caudal border for the T0 and T2 condylar volume, The T2 mandible was registered on the T0 mandible for each side separately using the left and right modified ramus as volume of interest using the same voxel-based registration procedure as stated above. This also allowed for the C-planes constructed on the T0 mandible to be used as the cut-off plane for the T2 mandible. The anterior lower facial and ramal height were used to calculate the A/P ratio (anterior/posterior lower facial height).

### Condylar resorption definition

To determine if a patient developed condylar resorption in this study, three conditions should be met: (1) volume loss of the mandibular condyle, (2) significant skeletal mandibular posterior displacement, and (3) loss in ramal height. Available literature states that at least 2 mm of posterior displacement^24^ and at least 2 mm of ramal height loss^25^ are to be regarded as clinically significant. There is less evidence on the amount of condylar volume loss. For this study, we accepted a net volume loss at T2 with a 30 mm^3^ segmentation error as a safety margin, as this has been found as a mean measurement error in condylar volumes^22^. Thus, a volume loss greater than 30 mm^3^, in combination with the two other conditions, was used to determine the presence of condylar resorption.

### Statistical analysis

Descriptive statistics on the cohort and the occurrence of resorption were calculated.

To identify risk factors, univariable analysis was performed on both patient level and condylar levels (n = 2 for each patient) since each ramus will have unique ramal height, condylar volume and surgical movements of the proximal fragment. For the univariable analysis, logistical regression was used. Since the data in the condylar level dataset is nested per patient (left and right side), generalized estimating equations (GEE) were used with an independent working correlation matrix to handle the potential patient clustering effect. *P*-values (α = 0.05), odds ratios and their confidence intervals were calculated. For the multivariable approach, machine learning models were trained and tested. Both XGBoost and Random Forest models were trained and tested on both datasets. The dataset was randomly split in a 70% training and 30% testing set. Due to the low number of patients that will develop condylar resorption, imbalanced data will occur with a majority of non-resorption patients. Synthetic Minority Oversampling (SMOTE) was applied to adjust for this imbalanced data on both data sets. All predictors were evaluated at the start. Predictor selection was performed based on feature importance. For the XGBoost a minimal importance score of ‘1’ was set for selection. For the predictor selection in the Random Forest model, Boruta analysis was used with 20 iterations and a *p*-value threshold of 0.05. The AUC, F-scores, Accuracy Rate, Misclassification rate, precision and recall of the model were reported and the best model was selected based on F-scores. All statistical analyses were done with Exploratory v9.2 (Exploratory Inc., USA, https://exploratory.io) except for the univariable analysis on a condylar level which was performed using SAS software v9.4 (SAS institute inc., USA).

### Shape analysis

The 3D models of the mandibles were used to compare the preoperative mandibular shapes of patients that developed condylar resorption with an age- and sex-matched control group of patients that did not develop resorption in the cohort. These were processed into a standard topology using the Meshmonk toolbox^26,27^ and statistical shape analysis was performed using custom-written MATLAB code. A partial least-squares regression of shape onto resorption status, following the protocol developed by Shrimpton et al., was used to test for significant differences between the groups^28^. Group differences were visualized as color maps. This analysis was done for the complete mandibular shape and left and right condylar and ramal shapes in bilateral resorption cases. For the patients with unilateral resorption and the respective affected left or right side of patients with bilateral resorption, the shape of the ramus and condyle were analyzed. The level of significance was set at 0.05.

## Results

Table 1 provides an overview of the characteristics of the 200 included patients. 19 patients (9.5%) met the criteria of condylar resorption, of whom 15 (7.5%) had a unilateral resorption, and 4 had a bilateral resorption (2%). On a condylar level, the resorption group on average had 230mm^3^ of volume loss of the condyle, which corresponds with an average volume loss of 17% . Their mandibles showed on average 3.9 mm ramal height loss and 3.1 mm of posterior translation of the distal segment due to the clockwise rotation caused by the ramal height loss. Table 2 provides an overview of the resorption determinants and differences between both groups on a condylar level.

**Table 1:** Demographics of the study population.

	Mean (sd)	Median [IQR]
n	200	
Age	24.5 (11.4)	19.0 [16.0, 30.3]
Sex		
Male (%)	84 (42)	
Female (%)	116 (58)	
Class		
II (%)	160 (80)	
III (%)	40 (20)	
BMI	22.1 (3.8)	21.3 [19.6, 24.2]
Preoperative mouth opening (mm)	47.0 (6.5)	46.0 [42.0, 51.0]
Preoperative TMJ pain		
No (%)	166 (83)	
Bilateral (%)	10 (5)	
Left (%)	15 (7.5)	
Right (%)	9 (4.5)	
Preoperative TMJ-sounds		
No (%)	122 (61)	
Bilateral (%)	25 (12.5)	
Left (%)	31 (15.5)	
Right (%)	22 (11.0)	
Procedure		
BSSO (%)	92 (46)	
BIMAX (%)	54 (27)	
BIMAX & Genioplasty (%)	27 (13.5)	
BSSO & Genioplasty (%)	27 (13.5)	
Mandibular Left–Right translation^a^ (mm)	− 0.3 (1.9)	0.0 [− 1.2, 0.9]
Mandibular Antero-Posterior translation^a^ (mm)	4.0 (5.0)	4.9 [0.8, 7.3]
Mandibular Up-Down translation^a^ (mm)	0.5 (2.9)	0.2 [− 1.2, 2.0]
Mandibular Pitch^b^ (°)	− 2.2 (4.2))	− 2.0 [− 4.5, − 0.2]
Mandibular Roll^b^ (°)	− 0.1 (1.5)	− 0.1 [− 1.0, 0.6]
Mandibular Yaw^b^ (°)	− 0.4 (2.1)	− 0.3 [− 1.4, 0.8]
Preoperative Left Condylar Volume (mm^3^)	1706.7 (537.2)	1652.2 [1338.9, 1989.9]
Preoperative Right Condylar Volume (mm^3^)	1712.9 (551.7)	1626.5 [1387.6, 2004.9]
A/P lower facial height ratio	1.2 (0.2)	1.2 [1.1, 1.3]
Mandibualr plane angle (°)	39.4 (7.9)	38.9 [33.7, 44.8]

*BMI* Body mass index, *TMJ* temporomandibular joint, *BSSO* Bilateral sagittal split osteotomy, *BIMAX* bimaxillary osteotomy, *A/P* Anterior/Posterior, *sd* standard deviation, *iqr* inter-quartile range.

^a^Positive values are right, anterior and upward translations.

^b^Positive values are clockwise rotations from respectively the right lateral, frontal and bottom view.

**Table 2:** Descriptive statistics of condylar resorption on a condylar/ramal level.

	Condylar Resorption (n = 23–5.7%)		No Condylar Resorption (n = 377–94.3%)		*p*
	Mean (sd)	Median [IQR]	Mean (sd)	Median [IQR]	
Mandibular posterior displacement (mm)	3.1 (0.9)	3.1 [2.3, 3.4]	0.60 (1.7)	0.7 [− 0.5, 1.6]	< 0.001*^a^
Vertical Ramal Height Loss (mm)	− 3.9 (1.7)	− 3.4 [− 4.6, − 2.7]	− 0.03 (2.2)	− 0.01 [− 1.3, 1.3]	< 0.001*^b^
Volumetric change (mm^3^)	− 230.2 (168.00)	− 209.0 [− 316.2, − 103.9]	− 48.8 (132.8)	− 33.1 [− 106.7, 31.6]	< 0.001*^b^
Volumetric change (%)	− 17 (11.4)	− 13.6 [− 24.8, − 7.8]	− 3 (8.41)	− 1.87 [− 6.5, 1.90]	< 0.001*^b^

*sd* standard deviation, *IQR* inter-quartile range, *p* *p*-value with α = 0.05.

*significant difference.

^a^A one-way anova was used for the normal variables.

^b^Kruskal-Wallis test for the nonnormal variables as found by a Shapiro–Wilk test.

Table 3 shows the descriptive statistics of the predictor variables on a patient level and the results of the univariable logistical regression. The odd ratio’s indicated an association between condylar resorption and a younger age, a bimaxillary + genioplasty procedure, larger mandibular advancements, upward movements of the distal segment, increased counterclockwise (CCW) pitch of the distal segment, smaller condylar volumes and higher anterior/posterior facial height ratios.

**Table 3:** Descriptive statistics of the resorption and unaffected group on a patient level (n = 200) with the results of the univariable logistical regression.

	Condylar Resorption (n = 19–9.5%)		No Condylar Resorption (n = 181–90.5%)		OR (95CI)	*p*
	Mean (sd)	Median [IQR]	Mean (sd)	Median [IQR]		
Age	18.58 (4.88)	17.00 [15.00, 20.00]	25.15 (11.72)	20.00 [16.00, 32.00]	**0.92 [0.85, 0.99]**	0.027*
Sex = Male (%)	5 (26.3)		79 (43.6)		0.46 (0.16–1.33)	0.153
BMI	22.00 (4.13)	21.26 [18.78, 24.63]	22.08 (3.73)	21.47 [19.58, 24.11]	0.99 [0.87, 1.13]	0.92
Preoperative Mouth Opening (mm)	46.68 (3.70)	47.00 [44.50, 50.00]	47.05 (6.69)	46.00 [42.00, 51.00]	0.99 [0.92, 1.07]	0.814
*Preoperative TMJ pain*						
No (%)	15 (78.9)		151 (83.4)		Base	Base
Bilateral (%)	0 (0.0)		10 ( 5.5)		0 (0-inf)	0.99
Left (%)	2 (10.5)		13 ( 7.2)		1.55 (0.31–7.52)	0.588
Right (%)	2 (10.5)		7 ( 3.9)		2.88 (0.55–15.1)	0.212
*Preoperative TMJ sounds*						
No (%)	11 (57.9)		111 (61.3)		Base	Base
Bilateral (%)	3 (15.8)		22 (12.2)		1.38 (0.35–5.34)	0.645
Left (%)	4 (21.1)		27 (14.9)		1.50 (0.44–5.06)	0.518
Right (%)	1 ( 5.3)		21 (11.6)		0.48 (0.06–3.92)	0.49
*Procedure*						
BSSO (%)	6 (31.6)		86 (47.5)		Base	Base
BIMAX (%)	2 (10.5)		52 (28.7)		0.55 (0.11–2.83)	0.476
BIMAX & Genioplasty (%)	7 (36.8)		20 (11.0)		**5.02 (1.52–16.56)**	0.008*
BSSO & Genioplasty (%)	4 (21.1)		23 (12.7)		2.49 (0.65–9.58)	0.18
Mandibular Left–Right translation^a^ (mm)	0.31 (1.61)	0.70 [− 0.80, 1.35]	− 0.35 (2.02)	0.00 [− 1.30, 0.80]	1.20 [0.92, 1.56]	0.17
Mandibular Antero-Posterior translation^a^ (mm)	9.29 (3.84)	9.90 [6.45, 11.90]	3.45 (4.81)	4.30 [0.50, 6.80]	**1.41 [1.21, 1.65]**	< 0.001*
Mandibular Up-Down translation^a^ (mm)	2.31 (4.08)	2.00 [− 0.15, 5.00]	0.25 (2.75)	0.00 [− 1.30, 1.80]	**1.24 [1.07, 1.44]**	0.005*
Mandibular Pitch^b^ (°)	− 4.95 (5.21)	− 5.00 [− 7.75, − 1.90]	− 1.86 (4.00)	− 2.00 [− 3.90, − 0.10]	**0.82 [0.73, 0.93]**	0.002*
Mandibular Roll^b^ (°)	0.19 (1.58)	0.30 [− 0.75, 0.75]	− 0.16 (1.45)	− 0.20 [− 1.00, 0.60]	1.18 [0.85, 1.64]	0.326
Mandibular Yaw^b^ (°)	− 0.45 (2.08)	− 0.40 [− 0.90, 0.65]	− 0.41 (2.15)	− 0.30 [− 1.40, 0.80]	0.99 [0.80, 1.24]	0.946
Preoperative Left Condylar Volume (mm^3^)	1413.24 (409.29)	1373.40 [1249.39, 1589.35]	1737.45 (540.61)	1677.48 [1389.77, 2021.35]	**0.99 [0.99, 0.99]**	0.013*
Preoperative Right Condylar Volume (mm^3^)	1445.61 (415.42)	1478.22 [1219.56, 1616.68]	1740.95 (557.63)	1664.44 [1425.59, 2019.14]	**0.99 [0.99, 0.99]**	0.027*
A/P lower facial height ratio	1.33 (0.21)	1.35 [1.20, 1.42]	1.22 (0.18)	1.20 [1.12, 1.31]	**13.58 [1.6, 115.1]**	0.017*
Mandibualr plane angle (°)	42.18 (9.22)	42.30 [34.52, 48.27]	39.05 (7.69)	38.68 [33.75, 44.73]	1.05 [0.99, 1.16]	0.102

*BMI* Body mass index, *TMJ* temporomandibular joint, *BSSO* Bilateral sagittal split osteotomy, *BIMAX* bimaxillary osteotomy, *A/P* Anterior/Posterior, *sd* standard deviation, *IQR* inter-quartile range, *OR* odd’s ratio, *95CI* 95% confidence interval, *p* *p*-value with α = 0.05.

*significant difference.

^a^Positive values are right, anterior and upward translations.

^b^Positive values are clockwise rotations from respectively the right lateral, frontal and bottom view.

This analysis was also performed on a condylar level. Table 4 provides the descriptive statistics of the predictor variables on a condylar level, together with the results of the univariable logistical regression with GEE. The same variables of the patient level were also confirmed to be associated with resorption in this analysis with the addition of an increased mandibular plane angle, a lateral, anterior and upward translation of the ramus and a CCW pitch of the ramus.

**Table 4:** Descriptive statistics of the resorption and unaffected group on a condylar level (n = 400) with the results of the univariable logistical regression.

	Condylar resorption (n = 23–5.7%)		No Condylar resorption (n = 377 − 94.3%)		OR (95CI)	*p*
	Mean (sd)	Median [IQR]	Mean (sd)	Median [IQR]		
Age	18.4 (4.5)	17.0 [15.5, 19.0]	24.9 (11.6)	20.0 [16.0, 32.0]	0.91 [0.86, 0.97]	0.001*
Sex = Male (%)	6 (26.1)		162 (43.0)		0.47 (0.18, 1.22)	0.119
BMI	20.9 (3.3)	20.5 [18.1, 21.7]	22.0 (3.7)	21.3 [19.5, 24.2]	0.91 [0.78, 1.06]	0.216
Mouth opening preop (mm)	45.9 (3.9)	45.0 [42.5, 49.5]	47.1 (6.6)	46.0 [42.0, 51.0]	0.97 [0.92, 1.02]	0.262
TMJ pain preop = TRUE (%)	6 (26.1)		62 (16.4)		1.79 (0.68, 4.73)	0.238
TMJ sound preop = TRUE (%)	10 (43.5)		146 (38.7)		1.22 (0.52, 2.85)	0.650
Procedure (%)						
BSSO (%)	7 (30.4)		177 (46.9)		Base	Base
Bimax (%)	2 (8.7)		106 (28.1)		0.48 (0.10, 2.34)	0.362
Bimax + genioplasty (%)	10 (43.5)		44 (11.7)		5.75 (2.07, 15.95)	< 0.001*
BSSO + genioplasty (%)	4 (17.4)		50 (13.3)		2.02 (0.57, 7.19)	0.276
Mandibular Left–Right translation^a^ (mm)	0.4 (1.7)	0.7 [− 0.8, 1.7]	− 0.3 (2.0)	0.0 [− 1.3, 0.8]	1.21 [0.93, 1.57]	0.153
Mandibular Antero-Posterior translation^a^ (mm)	9.6 (4.0)	9.9 [6.5, 12.7]	3.7 (4.9)	4.6 [0.5,7.0]	1.39 [1.22, 1.59]	< 0.001*
Mandibular Up-Down translation^a^ (mm)	3.0 (4.2)	3.3 [0.3, 5.2]	0.3 (2.8)	0.1 [− 1.3, 1.8]	1.3 [1.09, 1.55]	0.004*
Mandibular Pitch^b^ (°)	− 5.5 (5.3)	− 5.6 [− 10.1, − 1.9]	− 2.0 (4.1)	− 2.0 [− 4.2, − 0.1]	0.81 [0.69, 0.93]	0.004*
Mandibular Roll^b^ (°)	0.2 (1.5)	0.5 [− 0.8, 0.8]	− 0.1 (1.5)	− 0.2 [− 1.0, 0.6]	1.17 [0.86, 1.59]	0.316
Mandibular Yaw^b^ (°)	− 0.6 (2.2)	− 0.6 [− 0.9, 0.7]	− 0.4 (2.1)	− 0.3 [− 1.4, 0.8]	0.95 [0.77, 1.19]	0.676
Ramus Lateral translation^c^ (mm)	2.2 (1.2)	1.9 [2.8, 1.7]	1.6 (1.4)	1.6 [2.2, 0.9]	1.31 [1.08, 1.59]	0.008*
Ramus Antero-Posterior translation^a^ (mm)	2.1 (2.1)	2.4 [1.4, 3.5]	0.6 (2.3)	0.9 [− 0.4, 1.9]	1.52 [1.12, 2.07]	< 0.001*
Ramus Up-Down translation^a^ (mm)	2.0 (1.8)	1.6 [1.0, 2.5]	1.0 (1.8)	0.7 [− 0.1, 1.8]	1.30 [1.09, 1.54]	0.003*
Ramus Pitch^b^ (°)	− 6.5 (3.7)	− 7.0 [− 8.3, − 3.5]	− 2.9 (3.5)	− 2.8 [− 5.0, − 0.8]	0.75 [0.66, 0.86]	< 0.001*
Ramus Roll^b^ (°)	− 5.2 (3.4)	− 5.1 [3.0, 7.7]	− 4.7 (3.0)	− 4.5 [2.8, 6.7]	0.94 [0.80, 1.11]	0.474
Ramus Yaw^b^ (°)	− 1.9 (5.6)	− 1.0 [− 6.0, 1.6]	− 4.2 (3.7)	− 4.1 [− 6.6, − 1.4]	1.155 [0.98, 1.37]	0.093
Condylar volume preop (mm^3^)	1416.9 (358.7)	1363.7 [1243.4, 1589.4]	1727.6 (548.4)	1658.5 [1396.7, 2019.1]	0.99 [0.99–1]	0.001*
A/P height ratio	1.4 (0.2)	1.3 [1.2, 1.4]	1.2 (0.2)	1.2 [1.1, 1.3]	12.53 [1.58, 99.63]	0.017*
Mandibular plane angle (°)	44.0 (9.6)	43.1 [37.5, 52.2]	39.1 (7.7)	38.8 [33.7, 44.7]	1.08 [1.01, 1.16]	0.033*

^a^Positive values are right, anterior and upward translations.

^b^Positive values are clockwise rotations from respectively the right lateral, frontal and bottom view.

^c^For the Ramus inward translation, outward roll and yaw, the values of the right side were converted so they match the direction of movement of the left side perspective. Generalised Estimating Equations using an independent working correlation matrix were used to handle the potential patient clustering effect.

*BMI* Body mass index, *TMJ* temporomandibular joint, *BSSO* Bilateral sagittal split osteotomy, *BIMAX* bimaxillary osteotomy, *A/P* Anterior/Posterior, *sd* standard deviation, *IQR* inter-quartile range, *OR* odd’s ratio, *95CI* 95% confidence interval, *p* *p*-value with α = 0.05.

*significant difference.

**Fig. 2:**
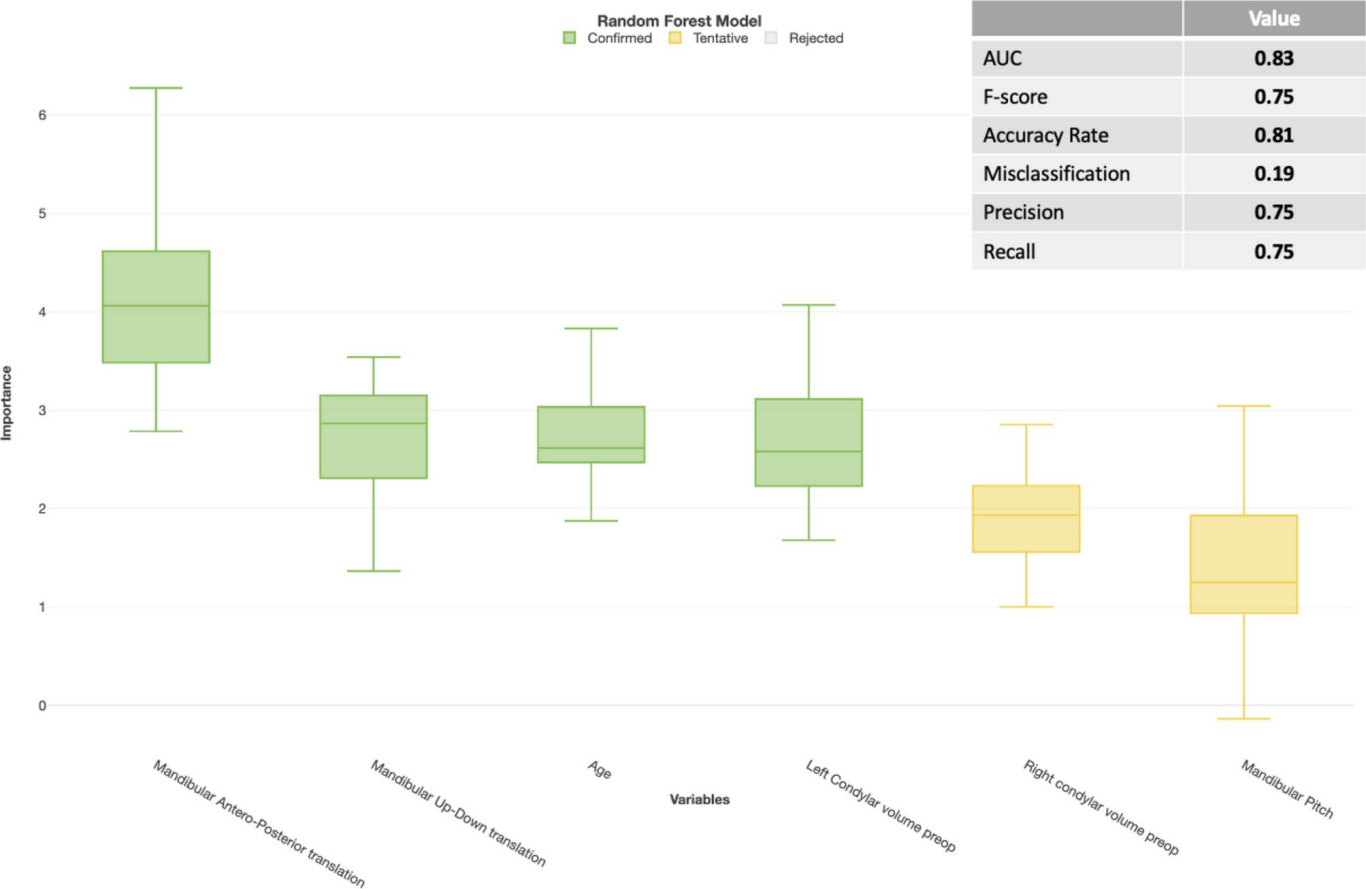
Importance analysis of Random Forest prediction model with predictor selection using Boruta analysis on a patient level. Decreasing importance of the variables from left to right. Model only contains certain or tentative predictors.

**Fig. 3:**
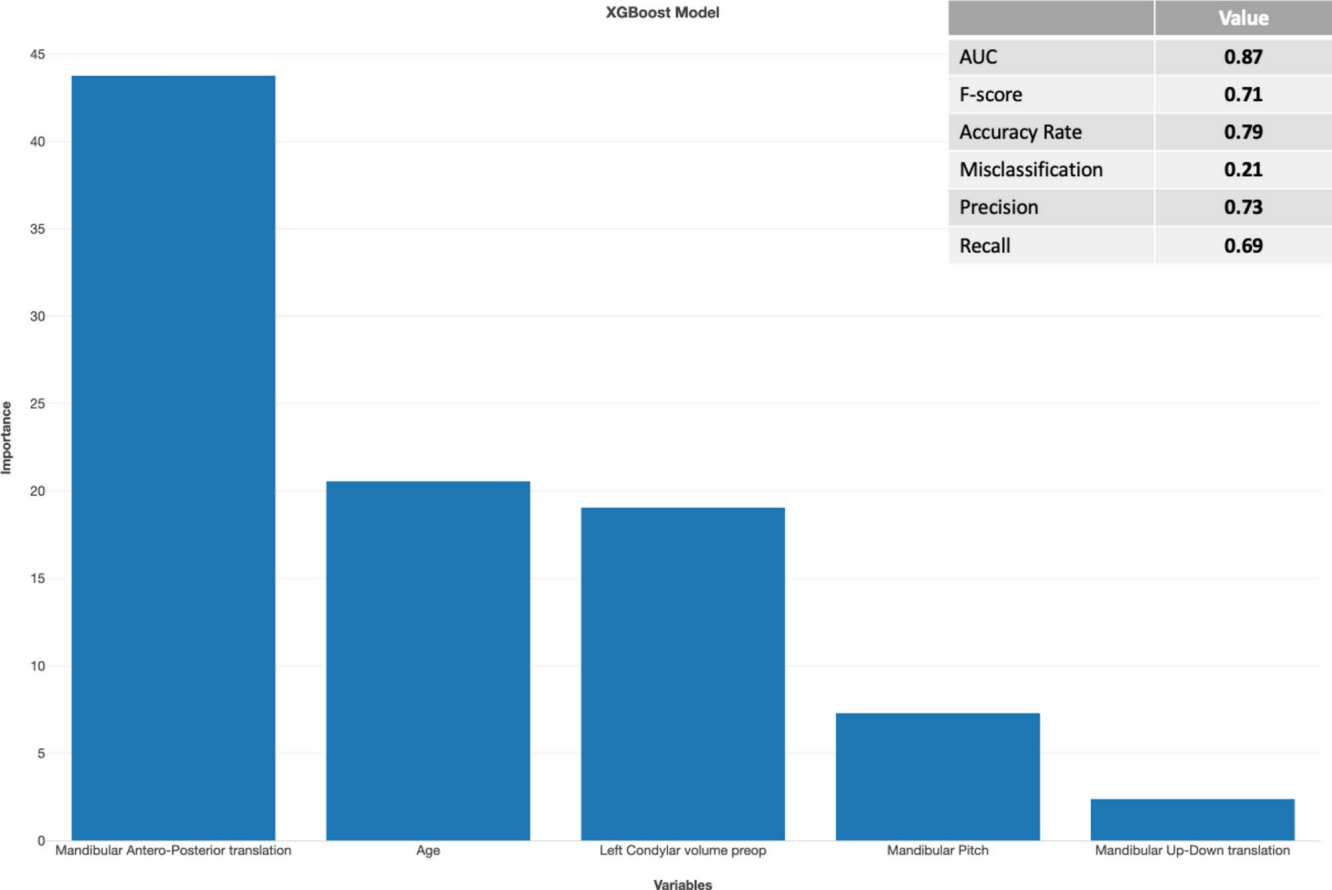
Importance analysis of the XGBoost prediction model on a patient level with predictor selection based on an importance score of 1 or higher. Decreasing importance of the variables from left to right.

**Fig. 4:**
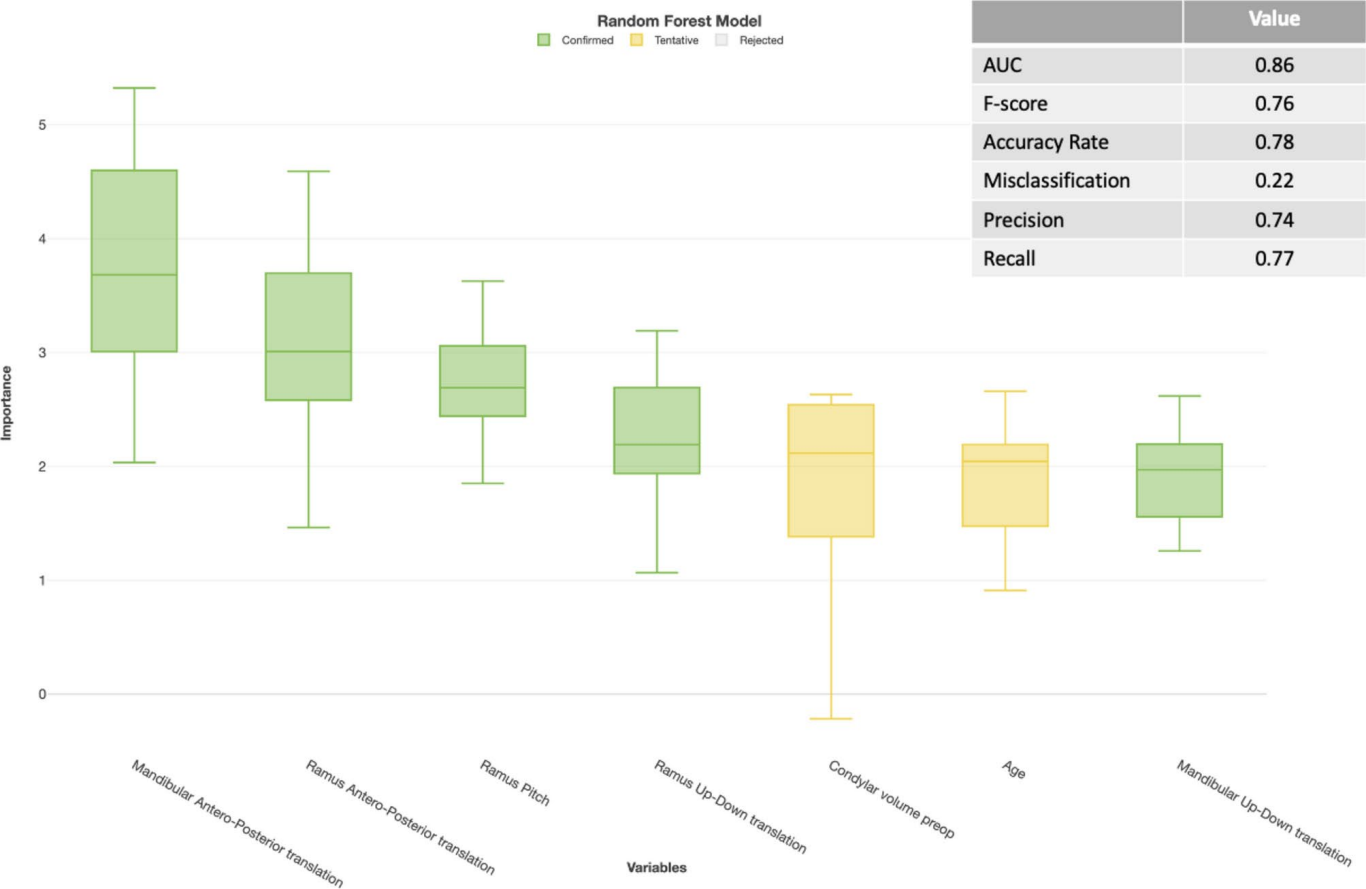
Importance analysis of Random Forest prediction model with predictor selection using Boruta analysis on a condylar level. Decreasing importance of the variables from left to right. Model only contains certain or tentative predictors.

**Fig. 5:**
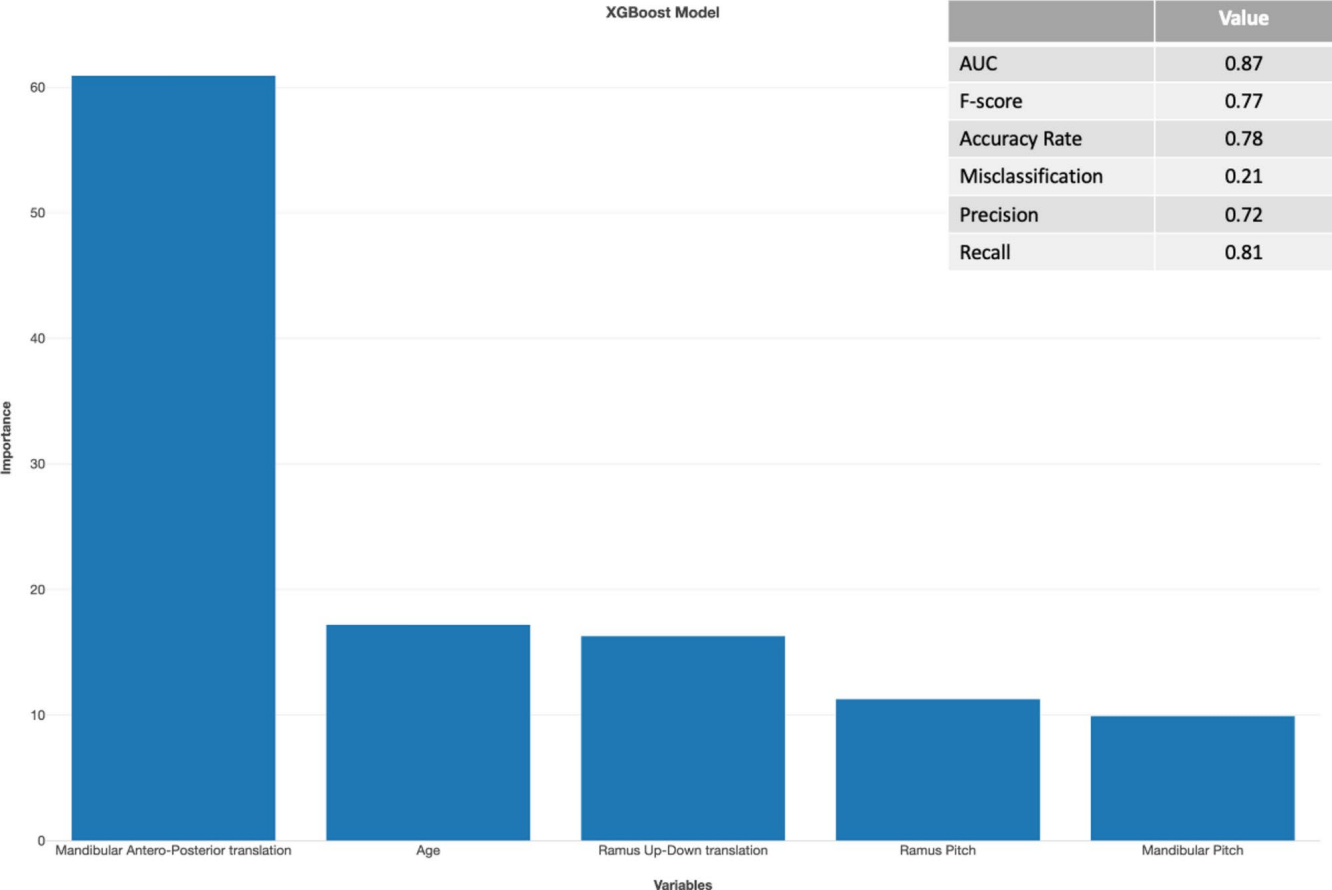
Importance analysis of the XGBoost prediction model on a condylar level with predictor selection based on an importance score of 1 or higher. Decreasing importance of the variables from left to right.

Results of the Random Forest and XGBoost machine learning models with predictor selection using respectively Boruta and importance scores are displayed in Figs. 2, 3, 4 and 5. For the patient level analysis, the Boruta (Fig. 2) and XGBoost (Fig. 3) were quite similar in performance. When looking at the F-score, the Random Forest model outperformed the XGBoost model with 0.75 versus 0.71. Boruta analysis allowed us to test for the importance of the explaining variables in this multivariable model and identified the amount of mandibular advancement, mandibular upward translation, a younger age, smaller condylar volumes and a CCW mandibular pitch as risk factors in the multivariable model with a good prediction performance. The XGboost model was a bit more stringent in the number of predictors, omitting right condylar volume and up-down mandibular translation.

For the condylar level analysis, the Boruta (Fig. 4) and XGBoost (Fig. 5) were even more similar in performance. In this case, the F score of the XGBoost model (0.77) was marginally better in comparison to the Random Forest model (0.76). Using the Importance analysis, we also identified the amount of mandibular advancement as the most important predictor for condylar resorption. A younger age, upward translation of the ramus, a CCW pitch of the ramus and mandible were also identified as risk factors in this multivariable prediction model.

**Fig. 6:**
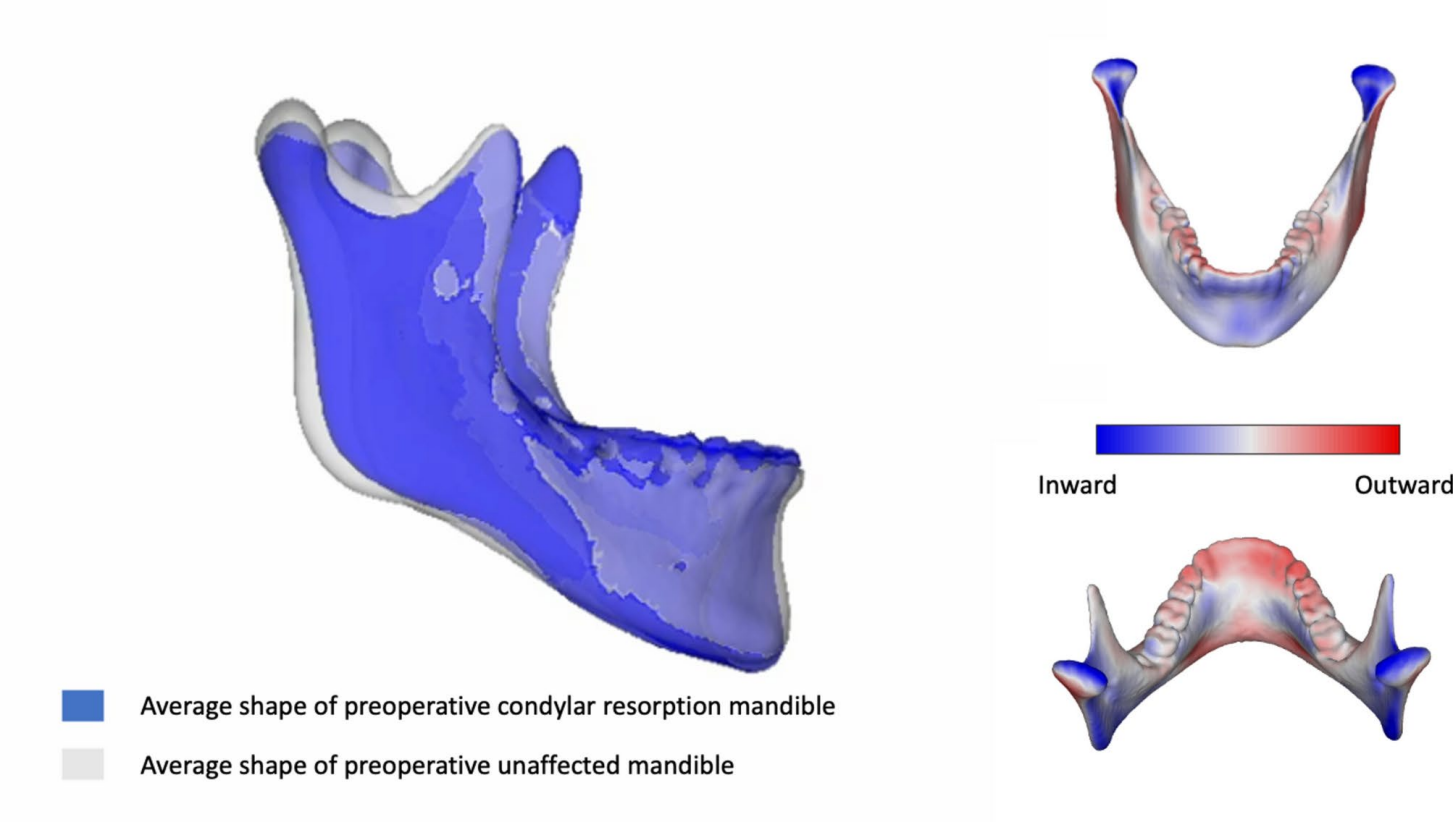
Shape analysis of full mandible in patients with bilateral condylar resorption. Mild shape differences are observed but without statistical significance.

For the patients with bilateral resorption (n = 4), no significant difference in shape was observed for the complete mandible (*p* = 0.10), left ramus (*p* = 0.06) and condyle (*p* = 0.8) and the right ramus (*p* = 0.38) and condyle (*p* = 0.14) when compared to patients without resorption. By inspecting the visual overlay in Fig. 6 (blue = resorption mandible), we can observe a suggestion of the identified smaller condylar preoperative volumes of patients that will develop resorption. However, the shape, which does not take size into account, is not found to be significantly different. Mandibular shape analysis did not show any significant differences in the unilateral analysis for the right (n = 6) ramus (*p* = 0.15) and condyle (*p* = 0.06) nor for the left (n = 17) ramus (*p* = 0.23) and condyle (*p* = 0.64).

## Discussion

The incidence of condylar resorption in the study cohort was 9.5%. This falls within the range found in the literature (between 1 and 31%) and below the 12.3% of a recent systematic review on the incidence of postoperative condylar resorption by Nino-Sandoval et al. in 2021^15,29^. Nonetheless, this is relatively high in contrast to clinical experience. These results show that one out of every ten patients having mandibular orthognathic surgery will experience condylar resorption to a certain degree. This is not always detected in routine clinical follow-up. Patients with an obvious problem will be diagnosed by their clinical symptoms, but a subclinical group exists for two reasons. First, in clinical follow-up, volumetric condylar analysis and imaging-based stability analysis are not performed routinely. This leads to detecting only severe cases evident to the clinician’s eye. Introducing easy, accessible postoperative analysis tools in virtual surgical planning software could remedy this. Second, postoperative orthodontics can camouflage occlusal characteristics of condylar resorption, such as an increased overjet or a mild anterior open bite. Patients who develop mild condylar resorption are at risk for a prolonged phase of postoperative orthodontics to compensate for these occlusal problems. Transparent communication in the case of unexpected prolonged postoperative orthodontics should help in detecting condylar resorption as well.

The condylar resorption group consisted of 19 patients, most with unilateral resorption. The average volume loss % in this group was 17%, which is the same as found in a previous study on condylar resorption by Xi et al.^11^, confirming the range of volume loss necessary to be labeled as condylar resorption. However, it should be clear that volume loss alone is insufficient to diagnose condylar resorption. For condylar volume loss to be labeled as condylar resorption, it should result in ramal height loss and posterior mandibular displacement. The combination of these conditions sets stringent diagnostic criteria for condylar resorption.

As for the patient profile, both class two and class three patients were included in the study. Although patients with mandibular hypoplasia are labeled as the classical patient at risk for resorption, condylar resorption can also occur in a minority class three patients.^30^ This study was set up to assess the occurrence and risk in a valid sample of patients presenting for orthognathic surgery at the author’s clinic, so a deliberate choice was made to also include class three patients as well. If condylar resorption occurs in a class 3 patient, this would not result in ‘relapse’ but in the emergence of a class 2 malocclusion. This study therefore does not speak of horizontal relapse but of horizontal posterior displacement of the distal segment as a criteria for resorption.

Risk factors were analysed on a patient level and a condylar level. This was done to properly investigate the effect of the proximal fragment as well. On a patient level, we confirmed previously found risk factors such as younger age, the amount of mandibular advancement, a CCW rotation of the mandible and a higher A/P lower facial height ratio^18,31,32^. We also identified novel risk factors such as bimaxillary surgery with genioplasty and mandibular upward movement as a risk factor. This last one contrasts with the downward displacement found in the study of Xi et al.^18^. These risk factors were reconfirmed on a condylar level, but we also gained insights into which surgical movements of the proximal fragment are associated with condylar resorption. Upward, lateral, and forward translation and CCW pitch rotation are associated with condylar resorption. In essence, these are all movements that cause compression of the condyle in the fossa. Compressive movements thus seem to be associated with the development of condylar resorption. Although it has been suggested in previous research^17,33^, this has never been confirmed by 3D assessment on a large scale. These results teach us to be aware of the role of the proximal fragment and its manipulation during condylar seating in the initiation of condylar resorption.

Machine learning prediction models allowed multivariable prediction modelling considering the confounding to assess the importance of risk factors. The advantages of machine learning are that it can handle datasets with many predictors and relatively few observations which was also the case in this study. The amount of mandibular advancement remains the most important risk factor in both models, which is in line with previous studies^17,18^. In the patient level prediction model, an upward translation of the mandible, a younger age, smaller preoperative condylar volumes and a higher mandibular CCW pitch were also confirmed to be highly important. When taking the proximal fragment into account, we see that younger age, an upward translation of the ramus, and a higher CCW mandibular and ramal pitch are of confirmed high importance in the prediction model. Female sex has been previously reported as a risk factor for resorption^9,14^. Our study did not confirm this finding. Five out of the 19 patients with resorption were males, with four developing unilateral resorption and one a bilateral resorption. These results show that postoperative condylar resorption does occur in males as well.

Shape analysis was performed to assess the hypothesis that mandibles or condyles that will develop resorption have a distinct shape, as suggested by Hoppenreijs and Hwang et al.^12,31^. Based on panoramic X-ray analysis, both studies found that a more posteriorly inclined condylar neck puts condyles at risk for resorption. Our 3D assessment did not confirm this finding. There were no statistical differences in the shape of the preoperative mandible, ramus or condyle of patients with or without resorption.

The clinical implications of this study highlight that condylar resorption occurs more frequently than previously expected. Clinicians should be aware that resorption varies in severity, with only the most obvious cases easily identifiable during chairside evaluations. Employing stringent radiological criteria, as recommended by this study, and routinely integrating quantitative imaging analysis could help detect subtle cases, particularly in patients undergoing prolonged postoperative orthodontic treatment to manage horizontal relapse, as previously discussed. Additionally, this study identified that specific movements of the distal and proximal segments, along with morphological factors like condylar volume and mandibular plane angle, are risk factors for resorption. These factors should be carefully considered when evaluating patients seeking combined orthodontic-surgical treatment or when planning orthognathic surgery using virtual surgical planning tools. These modern tools enable detailed planning, providing a comprehensive view of surgical movements in all six degrees of freedom (translation and rotation). When a surgeon anticipates large mandibular advancements or excessive rotations of the proximal fragment, it may be prudent to consider alternative surgical plans or ensure close postoperative monitoring of the temporomandibular joints.

This study has some limitations. Firstly, as there is no consensus on diagnostic criteria of condylar resorption, a pragmatic approach was used to construct these. The authors believe that a combination of the three proposed criteria creates an objectifiable diagnosis of condylar resorption with a stringent selection. Nonetheless, the method and cut-off values remain debatable. Secondly, we only used one year of follow-up to assess stability and condylar volumes. One could argue that this is insufficient and should be extended to two years, as done in the study on resorption by Xi et al. or even longer as done by Franco et al., who used three years of follow-up^18,34^. There is a chance that more patients with resorption will be diagnosed at two years of follow-up. The 1-year follow-up was chosen due to the availability of clinically relevant CBCT scans at this time point. Another limitation is the relatively limited amount of resorption cases in the cohort. This makes it difficult to properly design prediction models and to allow transfer of these findings to the general population of resorption cases. Results of the univariable analysis should be interpreted with caution. This was overcome in the machine learning model since the imbalanced data was corrected by using SMOTE. Nonetheless, the interpretation of these machine learning models also has their challenges. The same issue can be raised for the shape analysis. Patient numbers were low, limiting the power of the analysis. Finally, this study evaluated a single surgical protocol with the use of two miniplates as mandibular fixation. The effect of the choice of osteosynthesis technique on the proximal fragment and its role in condylar resorption can thus not be investigated.

## Conclusion

Condylar resorption occurs in 9.5% of patients having orthognathic surgery in this study, with the minority (2%) developing bilateral resorption. Patients with uni- or bilateral resorption had, on average, 17% volume loss of their condyles with 3.9 mm ramal height loss and 3.1 mm posterior displacement of their mandible one year after surgery. A younger age, a bimaxillary + genioplasty procedure, larger mandibular advancements, upward movements of the distal segment, a higher CCW pitch of the distal segment, smaller preoperative condylar volumes and a higher anterior/posterior lower facial height ratio were found to be risk factors based on univariable patient level logistic regression. Univariable analysis on a condylar level also identified compressive movements of the ramus and a higher mandibular plane angle as risk factors. Using machine learning prediction models for the multivariable analysis, the amount of mandibular advancement was the most important predictor for condylar resorption. Sex was not identified as a risk factor for postoperative condylar resorption. Preoperative mandibular, condylar or ramal shape was not different between patients with or without condylar resorption. These findings suggest that condylar resorption may be more common than previously thought. By identifying risk factors, surgical plans can be adjusted to reduce the likelihood of resorption, and patients can be more selectively screened for postoperative TMJ issues.

## Data Availability

The datasets generated and/or analyzed during the current study are not publicly available due to the GDPR regulation and hospital policy but are available from the corresponding author on reasonable request and after confirmation by our local ethical committee.
